# The Influence of Hypothyroid Metabolic Status on Blood Coagulation and the Acquired von Willebrand Syndrome

**DOI:** 10.3390/jcm12185905

**Published:** 2023-09-11

**Authors:** Manuela Andrea Hoffmann, Sarah N. Knoll, Pia-Elisabeth Baqué, Florian Rosar, Inge Scharrer, Stefan Reuss, Mathias Schreckenberger

**Affiliations:** 1 Department of Nuclear Medicine, University Medical Center of the Johannes Gutenberg-University, 55131 Mainz, Germany; sarahnknoll@gmail.com (S.N.K.); pia-elisabeth.baque@unimedizin-mainz.de (P.-E.B.); florian.rosar@uks.eu (F.R.); reuss@uni-mainz.de (S.R.); mathias.schreckenberger@unimedizin-mainz.de (M.S.); 2 Institute for Preventive Medicine of the German Armed Forces, 56626 Andernach, Germany; 3 Department of Nuclear Medicine, Saarland University Medical Center, 66421 Homburg, Germany; 4 Department of Haematology of the Medical Clinic and Policlinic, University Medical Center of the Johannes Gutenberg-University, 55131 Mainz, Germany; e.scha@t-online.de

**Keywords:** hypothyroid metabolic status, hypothyroidism, blood coagulation, hypocoagulability, acquired von Willebrand syndrome, bleeding risk, hyperfibrinolytic status

## Abstract

The intent of this prospective study aimed to identify the influence of hypothyroid metabolic status on the coagulation and fibrinolytic system and association with the acquired von Willebrand syndrome (VWS-ac). We compared 54 patients without substitution therapy after radical thyroidectomy with 58 control subjects without pathological thyroid-stimulating-hormone (TSH)-values. Patients with TSH > 17.5 mU/L over a period of >4 weeks were included. The control-collective was selected based on age and sex to match the patient-collective. The data were collected using laboratory coagulation tests and patient questionnaires; a bleeding score was determined. There were significant differences in the measurement of activated-partial-thromboplastin-time (aPTT/*p* = 0.009), coagulation-factor VIII (*p* < 0.001) and von-Willebrand-activity (VWF-ac/*p* = 0.004) between the patient and control groups. The patient cohort showed an increased aPTT and decreased factor VIII and VWF-ac. 29.7% of the patient-collective compared to 17.2% of the control subjects met the definition of VWS-Ac (*p* = 0.12). The bleeding score showed significantly more bleeding symptoms in patients with a laboratory constellation of VWS-ac (no family history; *p* = 0.04). Our results suggest hypocoagulability in hypothyroid patients. Hypothyroidism appears to have a higher incidence of VWS-ac. The increased risk of bleeding complications in hypothyroid patients may be of relevant importance for the outcome, especially in the context of invasive interventions.

## 1. Introduction

The effects of hypothyroidism on blood clotting were first documented in the 1960s [1]. However, the underlying mechanisms have not yet been conclusively clarified. Various proposed models are based on the influence of the synthesis of coagulation factors by thyroid hormones and on the interaction between autoimmune processes in the thyroid gland and hemostasis [2,3]. A bleeding tendency with general changes in the coagulation system and a strengthened occurrence with acquired von Willebrand’s syndrome have been described in hypothyroid patients [2]. In addition, hypothyroidism seems to be associated with both a hypocoagulable and a hypercoagulable state [4,5,6]. Gluvic et al. proposed that therapy-naïve subclinical and clinical hypothyroidism can cause a higher risk of cardiovascular disease (CVD) morbidity and mortality [7]. Xu et al. suggested a relationship of subclinical hypothyroidism with a prothrombotic state [6]. Hypothyroidism is the most common form of thyroid malfunction and is of great clinical relevance. About 99.5% of hypothyroidisms are primary hypothyroidisms and <0.5% have a central origin, i.e., the dysfunction lies in the pituitary gland or in the hypothalamus. The number of subclinical and manifest hypothyroidisms has increased in recent years, particularly in women [8]. Significant biochemical coagulation abnormalities and effects on fibrinolysis have been demonstrated in patients with manifest hypothyroidism. 

**Figure 1:**
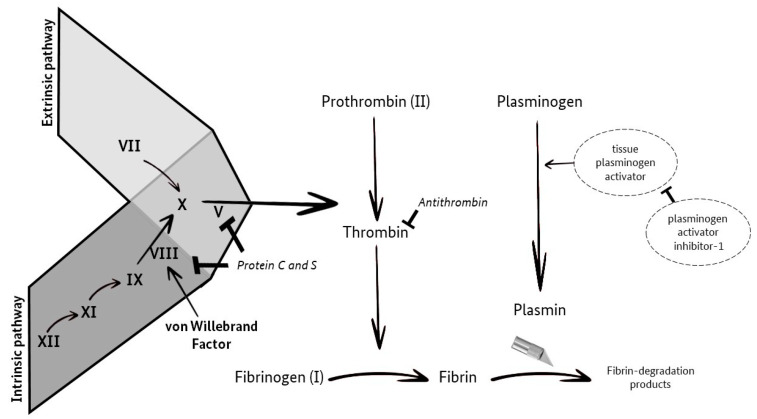
Coagulation and Fibrinolysis (own illustration, adapted from M. Krapick, based on Ellervik et al. [9]).

The aim of this prospective study was to investigate the possible connection between changes in the TSH value and the coagulation and fibrinolytic systems (Figure 1; [9]). Furthermore, our data were analyzed for a potential correlation of hypothyroidism with the occurrence of acquired von Willebrand syndrome (VWS-ac).

## 2. Materials and Methods

### 2.1. Patient and Control Collective

In this prospective cohort study, 54 patients with hypothyroidism (state after thyroidectomy without drug substitution) were analyzed and compared with control subjects. The prerequisite for inclusion in the patient group was a thyroid stimulating hormone (TSH) value > 17.5 mU/L over a period of more than four weeks. The selected patients were thyroid carcinoma patients with differentiated cancer, after radical thyroidectomy, from the Department of Nuclear Medicine at the University Medical Center in Mainz. According to the current guidelines for differentiated thyroid carcinomas, supplemental ablative radioiodine therapy is performed after thyroid surgery [10]. The efficacy of ablative radioiodine therapy depends on the TSH level. Patients should be in a manifest hypothyroid state at the start of therapy. For this purpose, no hormone substitution was administered for four weeks postoperatively until radioiodine therapy. At the time blood samples for this study were obtained, the patients had not received any hormone substitution with euthyroxine or L-thyroxine since surgical removal of the thyroid gland [10]. Therefore, manifest or latent hypothyroidism was assumed. The patients included in our study had no other diseases (such as Hashimoto’s thyroiditis or autoimmune thyreopathy Graves’ disease or other cancers) and no known distant metastases of thyroid carcinoma. Therefore, we assumed that no additional factors present in our patient population could cause coagulation disorders.

58 control subjects from the Gutenberg Health Study (GHS) served as the control collective. The GHS emerged from an interdisciplinary research project in cooperation with preventive cardiology and II. Medical Clinic and Policlinic of the University Medical Center Mainz [11] was initiated in 2007 at the University Medical Center Mainz. GHS is a population-based, prospective, monocentric cohort study in the Rhine-Main region. The first phase of the GHS ended in April 2017. The second phase of the study is expected to last until the end of 2027 and will include the 10-year follow-up study as well as the 15-year follow-up study. Study participants with a TSH value in the normal range (0.4–4.9 mU/L) were selected for the present study according to age and sex to match the patient group. 

### 2.2. Methods

The data were collected using laboratory coagulation tests and patient questionnaires. In addition, a bleeding score and a thrombosis score were determined.

For the coagulation tests, excess sample material from the outpatient follow-up check with routine diagnostics in the outpatient clinic of the Department of Nuclear Medicine at the University Medical Center in Mainz was used. In addition, the patients were asked about two symptom complexes and medication intake using a questionnaire. The pseudonymized patient information obtained from these questionnaires was then supplemented with further information from the patient files.

Questions on the following characteristics served as the basis for determining the bleeding score: increased nosebleeds, menstrual bleeding >4 days, prolonged bleeding or bleeding after dental treatment, bleeding gums, bleeding after surgery, general bleeding tendency and bleeding tendencies in the family, which also serve to improve the clinical diagnosis of von Willebrand disease (VWD) [12]. The following definitions of symptoms were given orally to patients:

- Nosebleeds: Any type of nosebleed that occurs daily or interferes with social activities.
- Skin hemorrhages: These were considered significant if ≥5 skin hemorrhages > 1 cm in size occurred at exposed sites.
- Bleeding gums: Prolonged bleeding in the mouth (>10 min) after minor injuries to the lip, tongue or cheek was counted.
- Tooth Extraction: Bleeding that occurs after leaving the dentist’s office, causing a delay in treatment or necessitating a repeat visit to the dentist.
- Bleeding after or during surgery: Bleeding reported by the surgeon as being unusually long or causing delays or special measures.
- Menstrual bleeding: Bleeding of a magnitude and duration that restricted daily activity, work or social life on most days of the menstrual period.

The thrombosis score consisted of questions about a potentially increased general susceptibility to thrombosis, family susceptibility to thrombosis, thrombosis after an operation, thrombosis during pregnancy, travel or immobilization or hospitalization. In addition, the body mass index (BMI) was calculated from the stated weight and height of the patients using the formula BMI = body weight in kg/height in m^2^. 

Blood samples were obtained in two citrate tubes with 3.2% sodium citrate solution and one tube with 3.8% citrate plasma from Saarstedt in Germany for the PFA-100 test. The surrogate parameters collected as part of the study and their reference ranges are listed in Table 1.

Thyroid stimulating hormone (TSH) is used for screening and for determining thyroid function in suspected malfunctions (hypothyroidism or hyperthyroidism) and for monitoring the progress of substitution therapy with thyroid hormone. In the present study, TSH was determined using the Architect TSH Assay (Abbott Wiesbaden, Germany).

The von Willebrand factor (VWF) is a glycoprotein that plays a central role in primary hemostasis. The specific von Willebrand factor antigen (VWF-Ag) content in the plasma was determined using a VWF-Ag test kit from Siemens Healthcare Marburg.

The test’s principle for determining the von Willebrand factor activity (VWF-Ac) is based on the binding of VWF to its glycoprotein Ib receptor. The INNOVANCE^®^ VWF-Ac particle-enhanced test from Siemens Healthcare Marburg was used to determine the VWF-Ac in this study.

Factor VIII is a glycoprotein and an important component of the endogenous coagulation system. The various coagulation factors VIII, IX, XI and XII were determined according to the identical test principle with the help of so-called deficient plasma. When carrying out the individual factor determination of factor VIII, the sample was mixed with the corresponding deficient plasma (lyophilized human plasma with a residual activity of factors VIII, IX, XI and XII of <1%) and then the activated partial thromboplastin time (aPTT) was measured. If factor VIII was missing in the sample, its absence in the deficient plasma cannot be compensated for. This results in prolonged aPTT. The activity of the coagulation factor in “% of the norm” was determined using a reference curve created with dilutions of standard human plasma or a normal plasma pool in combination with the corresponding deficient plasma. Factor VIII was determined if there was a suspicion of factor VIII deficiency in hemophilia A, VWS-ac, and/or inhibitor hemophilia to monitor therapy with factor VIII substitution. 

The Quick value is a laboratory parameter that provides information about the functional performance of the extrinsic coagulation system. The quick test is also known as the thromboplastin time (TPZ). The International normalized ratio (INR) was established in 1983 by the World Health Organization (WHO) as a standardization of the Quick value as part of improved monitoring of oral anticoagulation. The INR is calculated as follows: INR = TPZ patient plasma/TPZ normal plasma pool. The Dade^®^ INNOVIN^®^ reagent from Siemens Healthcare Marburg was used for this test in our study.

The aPTT is a global test that measures coagulation factors VIII, IX, XI, XII, precalli-crine and high molecular weight kininogen. The aPTT is also performed to monitor therapy with unfractionated heparin and perioperatively. Furthermore, the aPTT is a screening test used to detect congenital or acquired coagulation disorders (such as hemophilia A and B). A shortened aPTT can indicate increased coagulation activity. We measured aPTT using the Pathromtin SL reagent from Siemens Healthcare Marburg. This test identifies coagulation disorders in the endogenous system and measures factors VIII and IX and the contact factors. Internal quality control consisted of measuring two control samples in the normal range and in the pathological range. The reference range for the aPTT was between 26 and 36 s according to the reference range from the central laboratory of the University Medical Center Mainz (Swisslab).

We used the Platelet Function Analyzer (PFA) system (PFA-100) in vitro to determine primary hemostasis or platelet function. In the present study, the Dade^®^ PFA measuring cell collagen/Epinephrine (PFA-EPI) and Dade^®^ PFA measuring cell collagen/adenosine diphosphate (PFA-ADP) were used. In the PFA system, the process of platelet adhesion and aggregation is stimulated in vitro. PFA measuring cell collagen/EPI was primarily used to detect intrinsic thrombocyte dysfunction. For example, the collagen/ADP measuring cell was used to check whether the resulting abnormal results are due to medication containing acetylsalicylic acid. Here, a collagen-coated membrane replaces the function of the sub-endothelium in vivo in vitro. When the test is carried out, the thrombocytes attach to the collagen-coated membrane and the thrombocytes are activated by high flow velocities and shear forces, under the influence of which VWF can bind to the glycoprotein Iba receptor. Upon contact with the agonists EPI or ADP, the granule contents are released. With optical aggregometry, platelet function is assessed based on aggregate formation, which is determined by changes in transmitted light. Finally, the time taken for the membrane to completely occlude is determined.

### 2.3. Statistical Analysis

The statistical evaluation was carried out with the Statistical Package for the Social Sciences (SPSS) Statistics for Windows, Version 22.0 (IBM SPSS Statistics for Windows, Version 22.0, IBM, Armonk, NY, USA, 2013). A significance level of 0.05 was chosen for the statistical analyses. Continuous variables were examined for normal distribution using skewness measures and histograms. If the distribution was normal, a *t*-test was used. The Mann–Whitney U test was performed for non-normally distributed unrelated values and the Wilcoxon rank sum test for related non-normally distributed values. Potential influencing factors that had a *p*-value < 0.05 in the univariate analysis were then considered multivariate in a forward-looking linear regression. The confidence interval was 95%. For the descriptive part, the relative and absolute frequencies were calculated, and the mean with standard deviation (SD) and the median with inter-quartile range (IQR) were determined. Fisher’s exact test was used for the three variables and their combinations. The statistical evaluation was carried out after consultation with the Institute for Medical Biometry, Epidemiology and Computer Science (IMBEI) at the University Medical Center Mainz.

## 3. Results

### 3.1. Patient and Control Collective

The present patient collective consisted of 54 patients with TSH values > 17.5 mU/L and a mean TSH value of 73.34 mU/L (SD = 34.9 mU/L, reference range 0.4–4.9 mU/L). Of the patients in the patient collective, 40 (74.1%) were female and 14 (25.9%) were male. The patients were in a postoperative condition after thyroidectomy for thyroid carcinoma. They were recruited as part of their follow-up program. The mean age, regardless of sex, was 46.1 years (SD = 14.1 years). The age range was between 17 and 74 years. Nineteen patients (35.2%) were <40 years of age and eleven (20.4%) were ≥60 years of age. The male patients had a mean age of 50.4 years (SD = 11.7 years) and the female patients had a mean age of 44.5 years (SD = 14.6 years). The 54 patients had a mean BMI of 28.4 kg/m^2^ (SD = 7.4 kg/m^2^). One patient (1.9%) had a BMI in the underweight range (BMI < 18.5 kg/m^2^). Sixteen patients (29.6%) were normal weight (BMI 18.5–24.9 kg/m^2^), 20 patients (37%) were overweight (BMI 25–29.9 kg/m^2^), and 17 patients (31.5%) had obesity per magna (BMI ≥ 30 kg/m^2^).

The 58 control subjects in the present study showed TSH values in the reference range (0.4–4.9 mU/L) with a mean of 1.3 mU/L (SD = 0.9 mU/L). Of the control subjects, 42 (72.4%) were female and 16 (27.6%) were male. The age of the control subjects varied from 41 to 78 years. The mean age, regardless of sex, was 52.6 years (SD = 10.1 years). Fourteen patients (24.1%) were <40 years, and 14 patients (24.1%) were ≥60 years. The male subjects averaged 54.8 years (SD = 11.7 years), and the female subjects averaged 51.7 years (SD = 9.5 years) of age. The 58 control subjects had a mean BMI of 27.3 kg/m^2^ (SD = 5.1 kg/m^2^). In the control population, 21 control subjects (36.2%) were normal weight (BMI 18.5–24.9 kg/m^2^), 21 control subjects (36.2%) had excess weight (BMI 25–29.9 kg/m^2^), and 16 control subjects (27.6%) had obesity per magna (BMI ≥ 30 kg/m^2^).

### 3.2. Coagulation Status

**Figure 2:**
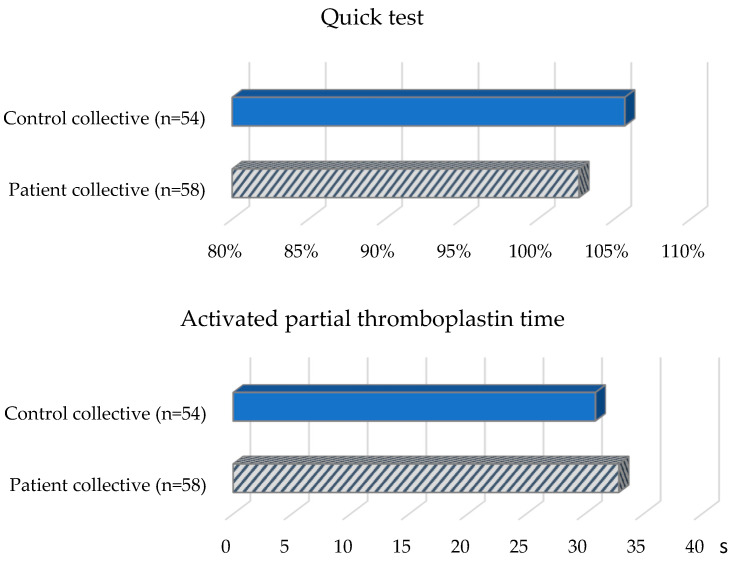

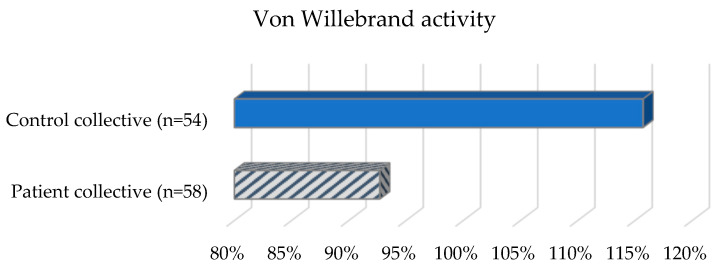
Coagulation status of the patient and control collective.

The patient collective (Table 2, Figure 2) revealed the following values with regard to the aPTT reference range (26–36 s): Nine (16.7%) patients had prolonged aPTT. 44 (81.5%) patients showed aPTT values in the normal range and one patient (1.9%) had a shortened aPTT. The mean value of all patients was 32.9 s (SD = 3.5 s). The Quick values of almost all patients in the group were in the reference range (70–120%). One patient had an elevated value. The mean value was 102.7% (SD = 11%). In the determination of VWF-Ag, five (9.3%) patients showed elevated values, 46 (85.2%) patients showed values in the normal range (50–160%) and three (5.6%) patients showed decreased values. The mean value of all patients in the group was 95.9% (SD = 38.7%). When VWF-Ac was determined, two (3.7%) patients had elevated values, 43 (79.6%) patients had values in the normal range (50–150%), and nine (16.7%) patients had decreased values. The mean value of all patients in the group was 92.7% (SD = 38.6%). Factor VIII was elevated in three (5.6%) patients, 48 (88.9%) patients had values in the normal range (70–150%), and three (5.6%) patients had decreased values. The median for all patients in the group was 106.5% (IQR = 38.9%). In the PFA-100 test, 33 (61.1%) patients showed an isolated or combined change: 17 (31.5%) patients showed an isolated increase in the PFA-EPI value (reference range: 84–160 s), and 19 (35.2%) patients showed no change or deviation. Six (11.1%) patients showed an isolated change in the PFA-ADP value (reference range: 68–121 s), of which three (5.6%) patients each showed a decrease or increase. Eleven (20.4%) patients showed an increase in both PFA-EPI and PFA-ADP. One patient (1.9%) showed a decrease in PFA-EPI and PFA-ADP.

Of the control subjects (Table 2, Figure 2) with TSH values in the reference range (0.4–4.9 mU/L), six (10.3%) subjects were shown to have a prolonged aPTT (reference range: 26–36 s). Forty-nine (84.5%) control subjects demonstrated aPTT in the normal range and three (5.2%) control subjects demonstrated a shortened aPTT. The mean aPTT was 30.9 s (SD = 4.4 s). All control subjects except one had normal Quick values (reference range: 70–120%). One subject showed an elevated Quick value. The mean value was 105.7% (SD = 9%). In the determination of VWF-Ac, 11 (19%) control subjects were in the elevated range, and 45 (77.6%) were in the normal range (50–150%); two (3.4%) control subjects showed decreased values. The mean value was 115.7% (SD = 43.9%). For factor VIII, 20 (34.5%) control subjects had elevated values and 38 (65.5%) control subjects had values in the normal range (70–150%). The median was 128.9% (IQR = 67.4%). In 32 (55.2%) control subjects, an isolated or combined change was detected when the PFA-100 test was performed: 19 (32.8%) control subjects showed an isolated increase in the PFA-EPI value. Twenty-six (44.8%) control subjects had neither an increased PFA-EPI nor an increased PFA-ADP value. Three (5.2%) control subjects showed an isolated elevation in the PFA-ADP value and ten (17.2%) control subjects showed an elevation in both PFA-EPI and PFA-ADP values.

### 3.3. Bleeding and Thrombosis Score

The evaluation of the questionnaires, which recorded the clinical symptoms of the patients, revealed the following abnormalities with regard to the bleeding score (Table 3). Of 54 patients, 13 patients reported abnormalities in the bleeding score. Seven (13%) patients reported one symptom and four (7.4%) patients reported two symptoms from the bleeding score. Two (3.7%) patients reported abnormalities in both the bleeding score and the thrombosis score (Table 3).

**Figure 3:**
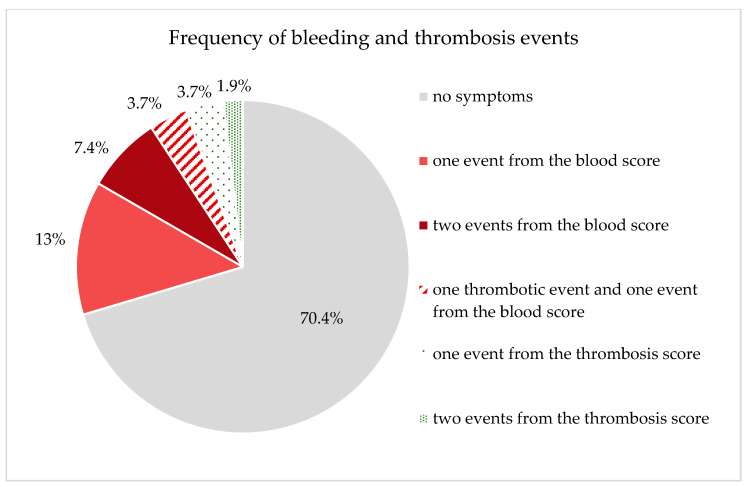
Graphically represents the patients’ responses regarding the frequency of bleeding and thrombosis events (due to the composition of the groups, the total sum cannot add up to 100%).

Of the 54 patients with completed questionnaires, five patients reported meeting one or two items from the thrombosis score (Figure 3). Two (3.7%) patients reported one event from the thrombosis score, one (1.9%) patient reported two different thrombotic events, and two (3.7%) patients described having observed one thrombotic event and one event from the blood score. Four (7.4%) of the five patients reported having a family history of known cases of thrombosis. Of these, one patient had a family background of bleeding symptoms and one patient had increased hematoma formation in himself. One (1.9%) patient reported both spontaneous thrombosis and thrombosis due to immobilization after bed rest. Unfortunately, no comparable data (bleeding and thrombosis score) were collected for the evaluation of the control population as part of the GHS.

### 3.4. Coagulation Tests

On average, the patients showed an aPTT of 32.9 s (SD = 3.5 s), and the control subjects showed an aPTT of 30.9 s (SD = 4.4 s). Analysis of the values using a t-test for independent samples revealed a statistically significant longer aPTT (t(110) = −2.66, *p* = 0.009) in the patient collective.

Patients had a mean VWF-Ac score of 92.7% (SD = 38.6%) and control subjects had a score of 115.7% (SD = 43.9%). When the values were analyzed using a *t*-test for independent samples, there was a statistically significant lower VWF-ac in the patient collective (t(110) = 2.94, *p* = 0.004). 

Regarding the Quick value, patients showed a mean Quick value of 102.7% (SD = 11%), and controls showed a mean Quick value of 105.7% (SD = 9%). An independent-samples *t*-test showed no statistically significant difference (t(102.78) = 1.55, *p* = 0.124) between the patient and control collectives.

The mean age of the patients was 46.1 years (SD = 14.1 years) and that of the controls was 52.6 years (SD = 10.1 years). Analysis of the values using a *t*-test for independent samples revealed a statistically significant difference (t(95.77) = 2.8, *p* = 0.006) between the two groups.

Patients had a mean BMI of 28.4 kg/m^2^ (SD = 7.4 kg/m^2^), compared with 27.3 kg/m^2^ (SD = 5.1 kg/m^2^) in controls. No statistically significant difference (t(93.1) = −0.93, *p* = 0.35) between the two groups was shown by the t-test for independent samples.

Statistically significant lower factor VIII levels (*p* < 0.001) were present in the patient collective after evaluation with the Mann–Whitney U test. 

The PFA-EPI values of the patient and control collectives did not differ statistically significantly after evaluation using the Mann–Whitney U test (*p* < 0.39). The same was true when comparing PFA-ADP (*p* < 0.97). The test results showed a statistically significant difference in factor VIII, VWF-Ac, and aPTT between the patient and control groups (*p* < 0.05). However, when evaluating these findings, it should be considered that the groups also differed statistically significantly with respect to age.

#### 3.4.1. Linear Regression

Furthermore, there were the following differences between the two groups (Table 4). The aPTT was on average 1.8 s longer in the patient group compared to the control collective. VWF-Ac was, on average, 16.4% lower in the patient collective compared with the control collective. Factor VIII showed a mean value 28.3% lower in the patient collective than in the control collective. The regression coefficient was for the group variable, with the control collective as the reference in all cases.

One of the aims of the present study was to clarify whether hypothyroidism can lead to the occurrence of VWS-ac. The criteria for VWS were as follows:

1. VWF-Ac < 50%;
2. PFA-EPI > 160 s + PFA-ADP > 121 s;
3. VWF-Ac < 50% + PFA-EPI > 160 s + PFA-ADP > 121 s.

**Figure 4:**
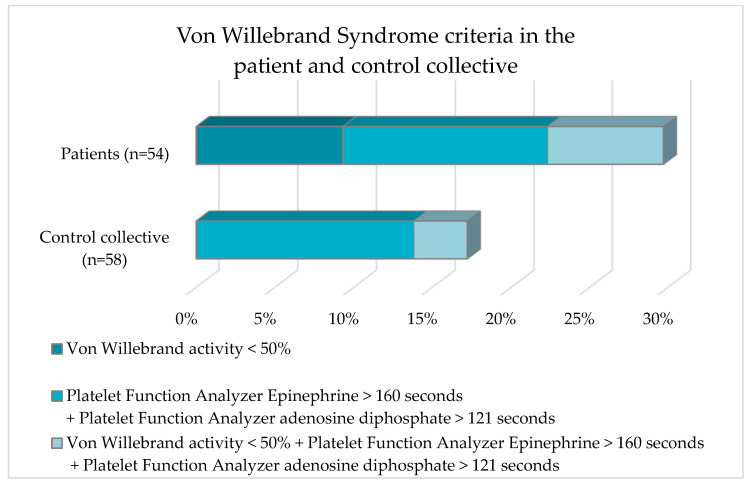
Frequency distribution of von Willebrand criteria of the patient collective and the control collective.

The frequency distribution of these characteristics in the two collectives is shown in Table 5 and Figure 4.

Five subjects in the patient collective had a VWF-Ac < 50%. No subject in the control collective was affected. A PFA-EPI > 160 s + a PFA-ADP > 121 s was shown by seven and eight subjects in the patient and control collective, respectively. The laboratory parameter constellation VWF-Ac < 50% + PFA-EPI > 160 s + PFA-ADP > 121 s occurred approximately twice as frequently in the patient collective as in the control collective (7.4% vs. 3.4%). Analysis of the data with Fisher’s exact test revealed no statistically significant difference (*p* = 0.12) for the occurrence of VWS in the comparison between the patient collective and the control collective. 

**Figure 5:**
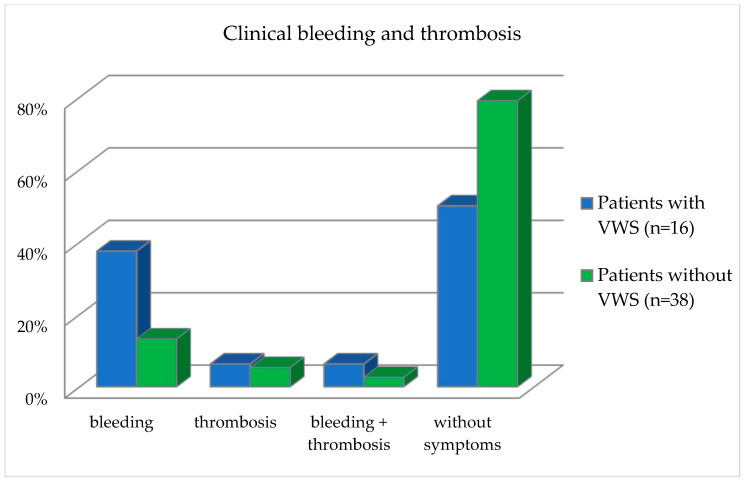
Frequency distribution of clinical bleeding and thrombosis events in the patient collective divided into patients with and without VWS.

Furthermore, the clinical bleeding and thrombosis events recorded in the questionnaires of the patient group were divided into patients with and without VWS (Table 6 and Figure 5).

Patients in the bleeding category reported having observed one or two bleeding events. Patients in the thrombosis group reported observing one or two thrombotic events. Patients in the category bleeding and thrombosis reported having observed both a thrombotic and a bleeding event. With regard to the occurrence of bleeding, the patients differed statistically significantly (*p* = 0.04), divided into the presence of VWS and without VWS, as calculated with the Fisher exact test (Table 6 and Figure 5).

#### 3.4.2. Combined Analysis of Questionnaires and Laboratory Values

##### Patient Collective

Only medications relevant to bleeding and thrombosis tendency were reported and considered (Table 7).

Of the patients with VWS, two were taking anticoagulant medications, and these also reported bleeding events. The other patients were not taking any medication that could explain their symptoms. Of the patients with a laboratory constellation not suggestive of VWS, one patient was taking an oral contraceptive and reported one thrombotic and one bleeding event. One patient was taking Prasugrel (Efient^®^) regularly and reported one bleeding event. Of the patients with VWS, none were taking medications that could have caused the VWS.

##### Control Subject Collective

Only medications relevant to bleeding and thrombosis tendency were reported and considered (Table 8). No data on bleeding or thrombosis score were available for the control subjects.

##### Evaluation of the Medication History (Table 9)

Among the drugs that can trigger VWS, we included in particular the use of valproic acid. We included drugs, such as acetylsalicylic acid (ASA) and other platelet aggregation inhibitors (e.g., Plavix^®^), direct and indirect oral anticoagulants, heparin, and selective serotonin reuptake inhibitors (SSRIs), among those that may cause prolongation of bleeding time. In particular, we evaluated oral contraceptives and other hormonal preparations as drugs that promote the occurrence of thrombosis.

## 4. Discussion

The influence of thyroid hormones on the coagulation and fibrinolytic system and its underlying mechanisms has not been conclusively clarified. The aim of this prospective study was to investigate a possible correlation between an elevated TSH and changes in the laboratory parameters VWF-Ag, VWF-Ac, factor VIII, Quick test, aPTT, PFA-EPI and PFA-ADP as well as to analyze a possible correlation with the occurrence of the acquired VWS. A sub-study objective of this prospective cohort study was to find out whether bleeding or thrombosis tendencies and acquired VWS were more frequent in patients with elevated TSH levels than in the control collective. For this purpose, 54 patients after radical thyroidectomy without substitution therapy were compared with 58 control subjects without pathological TSH levels. In order to determine whether the patients had noticed symptoms indicating a bleeding or thrombosis tendency, a bleeding and thrombosis score was collected in the patient collective. These data were also analyzed in conjunction with laboratory results for the diagnosis of VWS-ac. In addition, a medication history was obtained from both patients and control subjects to determine whether any of the medications taken may be associated with bleeding or trigger VWS. 

Concerning the bleeding tendency, statistically significant differences were found between the patient and control populations when evaluating the laboratory values obtained, but not for the Quick value (*p* = 0.124), PFA-EPI (*p* < 0.39) and PFA-ADP (*p* < 0.97). There were statistically significant differences in the measurement of aPTT (*p* = 0.009), VWF-Ac (*p* = 0.004) and factor VIII (*p* < 0.001). All three values showed a tendency towards a hypocoagulative state in the patient collective. Thus, aPTT was found to be prolonged in the patient collective compared with the control subjects. VWF-Ac and factor VIII were decreased compared to the control group. These differences represent the association of hypocoagulation in patients with a TSH > 17.5 mU/L. Recent studies support the thesis that a hypothyroid hormone state is associated with hypocoagulability and hyperfibrinolysis [2,13]. Our results are in line with a prospective study that also showed statistically significant increased aPTT (*p* < 0.001) and statistically significant decreased VWF activity and factor VIII (*p* < 0.001) in hypothyroid patients with a history of total thyroidectomy [14]. The authors compared three different metabolic patient situations: hypothyroidism, euthyroidism and patients given recombinant human TSH (rhTSH). The supply of exogenous TSH showed no influence on the clotting criteria. The study results indicated that thyroid gland hormone deficiency is probably the main source of clotting disorders in hypothyroid patients [14]. 

The literature to date suggests that, on the one hand, hypothyroidism can lead to an increased risk of bleeding. On the other hand, even low FT4 values that are still within the reference range can lead to a heightened bleeding risk [15,16]. However, the study of Elbers et al. could not verify a higher danger for considerable bleeding in patients with low FT4 levels following bariatric surgery [15], while in these weight reduction surgeries bleeding is one of the most important postoperative complications to consider [2,15]. However, the authors acknowledged that the patients included in the study had an increased baseline risk of hypercoagulability and venous thromboembolism due to their obesity [15,17].

Chadarevian et al. reported that patients with marked manifest hypothyroidism with TSH levels > 50 mU/L tend to have hypocoagulative states with increased fibrinolytic activity, whereas patients with moderate manifest hypothyroidism (TSH 10–50 mU/L) tend to have hypercoagulative states with decreased fibrinolytic activity and high risk for CVD [18]. This distinction could not be confirmed in the present study, including patients with a TSH > 17.5 mU/L. According to Ordookhani et al., a hypocoagulative status is associated with an overt hypothyroid metabolic state, whereas subclinical hypothyroidism is connected with a prothrombotic state [5]. Apart from that, a large population-based cohort study with 5918 participants showed a pro-coagulant state at elevated or highly normal FT4 levels. A link to cardiovascular risk factors was not found. However, VWF and fibrinogen arranged up to 10% of the connection between FT4 and CVD [19]. 

An increased risk for CVD has also been reported [7]. The authors showed an association of a therapy-naïve subclinical and clinical hypothyroidism and a higher risk for CVD morbidity and mortality [7], but add that this is one of the most discussed thyroid-related phenomena. Additionally, Inoue et al. analyzed data based on a nationally representative database of US adults [20]. The authors showed a connection between CVD and TSH values in the upper normal range and subclinical hypothyroidism with all-cause mortality, but no association was seen in patients with low TSH levels [20]. In our study, definitive data were not available for all patients and control subjects (no full set of data of follow-up for the incidence of CVD process or cardiovascular death), which would have allowed a statistical evaluation of cardiovascular risk. Xu et al. suggested a relationship between subclinical hypothyroidism and a prothrombotic state and recommended regular screening checks for cardiovascular risk factors as part of thyroid hormone monitoring [6]. 

Studies in recent years have highlighted the importance of an accurate history of clinical bleeding symptoms in diagnosing a bleeding tendency [21]. The “Bleeding Assessment Tool” for a VWD distinguishes between normal and abnormal bleeding and is also helpful in identifying patients with mild bleeding and describing the severity of bleeding disorders [12]. In order to take into account the clinical aspects of the diagnosis of a bleeding disorder, the patient group in the present study was interviewed by means of a questionnaire regarding important symptoms that occur in the context of a bleeding tendency. The bleeding score that we used took into account the symptoms previously described as important [22]. Only our subjects from the patient collective completed the questionnaire; corresponding data for the control collective were not available. A comparison of the two groups in terms of clinical symptoms was not possible.

An incidence of bleeding events not to be classified as serious was reported to be 5.3 and 7.7 per 100 persons per year by the Thrombosis Prevention Trial and the Women’s Health Study [23,24,25]. In the present study, 13 of the 54 patients reported mild bleeding events. The incidence of severe bleeding symptoms is close to zero in the general population, with trivial bleeding events presumably much more common than severe ones [23,24,25,26]. Overall, our patient population showed more frequent non-major bleeding symptoms (as defined by Rodeghiero et al. [22]) than healthy control subjects in other studies [23,24,25,26]. It should be mentioned here that the bleeding score collected in our study cannot be directly compared with those of other studies because the questionnaires differ with respect to the time period asked and the intensity of bleeding events.

When bleeding events occur, the differentiation between patients with and without coagulation system disease is still a major challenge for the practitioner in the context of the classification and diagnosis of bleeding symptoms. This also applies to the present work. In this context, it must be taken into account that an increasing number of people are taking anticoagulant medications, especially platelet aggregation inhibitors, vitamin K antagonists and direct oral anticoagulants (DOACs) [27]. This tendency is also reflected in the present patient population. The incidence of mild to moderate bleeding events is expected to be only marginally higher in patients with VWD type 1 and in patients taking aspirin compared to healthy subjects. In patients taking vitamin K antagonists, the study evidence suggests varying rates of bleeding. Severe subtypes of VWD are associated with higher rates of bleeding events [25,28,29,30]. For this reason, it was important for our study to obtain a medication history of patients with bleeding symptoms. Of the patients who reported bleeding symptoms, only three patients were taking a platelet aggregation inhibitor. Thus, the medication bias of the results can be excluded.

Due to the exponentially rising bleeding risk with increasing age, it was important to take age into account in the evaluation of our study [25]. Our patient collective with bleeding symptoms was, on average, 38 years old. Therefore, for study purposes, there was no high average age in the patient collective, which might otherwise have led to a bias in the results. However, the overall comparative analysis should consider that, despite the matching of the patient and control collectives, significant age differences (mean value ± standard deviation: 46.1 ± 14.1 vs. 52.6 ± 10.1) were found between the groups. Based on these results, age was included in the linear regression for adjustment. However, the patient population had a very similar distribution in terms of sex and age with respect to the general population at the onset of thyroid cancer. Of the patients, 40 (74.1%) were female and 14 (25.9%) were male. The males had a mean age of 50.4 years (SD = 11.7 years) and the females had a mean age of 44.5 years (SD = 14.6 years). According to the Robert Koch-Institute 3954 women and 1830 men in Germany were diagnosed with thyroid cancer in 2019. The median age of onset in the general population was 51 for women and 56 for men, which is relatively low compared to other cancers [31]. In this study, 92.3% of the patients who had observed bleeding symptoms in themselves were female. The literature also describes a higher incidence of bleeding complications in females compared to males due to sex-specific problems (e.g., heavy menstruation, bleeding during childbirth or during pregnancy) [25,32]. Our screening questionnaires asked about prolonged period bleeding or postpartum bleeding. A statistical analysis of the sex-specific differentiation was not possible in the present study due to the significantly higher proportion of women and the low number of cases. 

Our data indicated that many of the patients with abnormal lab values did not report any bleeding symptoms. One possible explanation is that the patients did not consider particularly mild bleeding events worth mentioning. Another possibility is that, despite changes in laboratory values, bleeding symptoms had not yet manifested themselves. Abnormal laboratory values in the absence of a clinical correlate are a frequently observed constellation in VWS. Therefore, the findings of the present evaluation indicate a possible association of VWS-ac with hypothyroidism.

Furthermore, the present work aimed to clarify whether there is an association between elevated TSH levels and VWS-ac. Several studies have described a strengthened increased occurrence of acquired von Willebrand’s syndrome in hypothyroid patients [2,16]. In a randomization genetic study by Ellervik et al. that included 17,558 hypothyroid participants and 117,083 controls, the authors concluded that genetically raised TSH was linked with degraded VWF levels [9]. Overall, 29.7% of the patient population and 17.2% of the control subjects in this study fulfilled the definition of the VWS-ac. However, this difference was not statistically significant (*p* = 0.12).

Stuijver et al. demonstrated a prevalence of VWS in 33% of patients with hypothyroidism using a comparable study design. They distinguished between those with a severe form of acquired VWS-ac (VWF-Ag and/or VWF-RCo ≤ 10%), a moderate form (VWF-Ag and/or von Willebrand Factor Ristocetin Cofactor Activity/VWF: RCo 10–30%), and a mild form (VWF-Ag and/or VWF-RCo 30–50%) of VWS-ac. In the present work, only mild forms of VWS occurred according to the definition of Stuvijer et al., whereas they diagnosed a moderate form in 9% of their patients and a mild form of VWS in 23%. The mean VWF-Ac/VWF-Ag ratio was nearly 1.0 in the present patient population, comparable to that of Stuijver et al. with 1.1 (reference range: 0.7–1.5), which suggests a quantitative VWF deficiency rather than a molecular defect. The authors, supported by other studies, were also able to demonstrate significantly increased VWF values in almost all study patients after the patients could be brought into a euthyroid metabolic state [2,14,16]. 

In the present study, it was striking that also in the control collective many subjects showed both PFA-EPI and PFA-ADP time prolongation. There are several possible explanations for this finding. Congenital VWD is one of the most common hemorrhagic diatheses, with a prevalence of 1% in the population [33]. Thus, there is a high probability that probands in the control population were affected by this disease. Another potential explanation for the increased incidence of PFA-ADP and PFA-EPI time changes is the use of drugs that affect blood coagulation. However, no medications that could have triggered VWS-ac were recorded in the subjects of the control collective, who were abnormal with respect to coagulation parameters. However, it cannot be ruled out that any underlying diseases of the control subjects (subcollective of the GHS, a cross-sectional survey of the population) were not known or not indicated and were therefore not considered in the analysis [11]. In this context, chronic diseases associated with the VWS-ac, such as malignancies, renal failure, diabetes mellitus, liver disease, arteriosclerosis and infections [34] could be considered. Since nicotine consumption may cause a slightly prolonged occlusion time of vessels and capillaries, a smoking history might provide another explanation [35]. Of the patients with abnormal laboratory findings, 43.8% reported bleeding symptoms. Stuijver et al. give a possible explanation for the lack of perception of bleeding symptoms, here in more than half of the patients, due to the mild expression of symptoms occurring in the context of VWS [16]. However, it was noticeable that our patients with a laboratory constellation of a VWS reported significantly more bleeding symptoms (*p* = 0.04) than the patients without these lab results. None of the patients with an abnormal laboratory constellation in this study had bleeding events in the family history; this might be interpreted as an indication of a VWS-ac [36].

According to the literature, neoplasia can cause adsorption of VWF to malignant cell clones or other cell surfaces and induce specific or non-specific autoantibodies that lead to increased degradation of VWF. Studies recommend that when a bleeding tendency appears in a serious disease process (e.g., myeloproliferative syndrome), acquired VWD must always be taken into account. However, cancer is generally associated with a change in hemostasis, leading to a prothrombotic state [37]. This aspect was not considered further in our study evaluation since the thyroid carcinoma had already been surgically removed in the included patients and our patients had no evidence of other carcinomas; thus, a complete cure could be assumed. Other studies suggest that after the healing of a neoplastic disease, a VWS-ac is often no longer detectable. Nicol et al. stated that long-term remission of VWS-ac can be reached by the treatment of the underpinning clonal cells of lymphoid tumors [38,39].

There are several limitations to our prospective study. One limitation is that a bleeding and thrombosis score could not be additionally collected from the subjects in the control group because this collective contained individuals who were screened as part of a survey to map the general population of the GHS. No definitive data were available for a statistical evaluation of the cardiovascular risk in the patients compared to the control population; however, it was not one of the study objectives to analyze whether an association of thyroid function and CVD is mediated by coagulation factors. Another limitation of the study was that no information on blood group affiliations was available for the study participants. This information could have been important, since blood group 0 can be associated with 25–30% lower VWF values [40].

## 5. Conclusions

Our results suggest a hypocoagulable state in patients with hypothyroidism. Hypothyroid function appears to have a higher incidence of VWS-ac. Bleeding tendencies were more frequent in patients with a laboratory constellation of VWS than in patients without this. 

Increased bleeding risk complications become particularly essential during planned invasive surgical procedures for the outcome of hypothyroid patients. Early bleeding prophylaxis in the context of well-controlled patient management, especially of laboratory parameters indicating hypothyroidism and altered VWS values, is important. Treatment should also aim to harmonize the risk of bleeding and thrombosis. Furthermore, when bleeding events of unclear origin occur, a hormonal control disorder of the thyroid gland should always be considered, and the corresponding laboratory diagnostics should be initiated.

Further studies with larger patient groups and repeated measurements are recommended in order to determine whether there is an adaptation with normalization of the coagulation parameters or an aggravation in the course of hypothyroidism, as well as for a detailed differentiation of patients with VWS-ac. Complementarily, it would be interesting to study coagulation parameters and other factors influencing coagulability in subclinical hypothyroidism.

## Figures and Tables

**Table 1:** Laboratory parameters with reference range of the central laboratory of the university hospital Mainz.

Parameters	Reference Rage	Unit
Thyroid stimulating hormone (TSH)	0.4–4.9	mU/L
Von Willebrand factor antigen (VWF-Ag)	50–160	%
Von Willebrand factor activity (VWF-Ac)	50–150	%
Factor VIII	70–150	%
Quick test	70–120	%
International normalized ratio (INR)	0.85–1.27	
Activated partial thromboplastin time (aPTT)	26–36	s
Platelet Function Analyzer adenosine diphosphate (PFA-ADP)	68–121	s
Platelet Function Analyzer epinephrine (PFA-EPI)	84–160	s

**Table 2:** Sociodemographic data, patients’ characteristics and coagulation parameters of the patient and control collective.

	Patient Collective	Control Collective
	(*n* = 54)	(*n* = 58)
	Mean Value ± Standard Deviation (SD)	Mean Value ± SD
Age (years)	46.1 ± 14.1	52.6 ± 10.1
Weight (kg)	82.2 ± 23.8	78.1 ± 2.0
Body size (m)	1.70 ± 0.1	1.70 ± 0.1
Body mass index (kg/m^2^)	28.4 ± 7.4	27.3 ± 5.1
Differentiated thyroid cancer	54	0
Distant metastases (thyroid cancer)	0	0
Other cancers	0	0
Chemotherapy	0	0
Graves’ disease or Hashimotothyroiditis	0	0
Quick test (%)	102.7 ± 11	105.7 ± 9
aPTT (s)	32.9 ± 3.5	30.9 ± 4.4
VWF-Ag (%)	95.9 ± 38.7	
VWF-Ac (%)	92.7 ± 38.6	115.7 ± 43.9
	Median/interquartile range (IQR)	Median/IQR
Faktor-VIII (%)	106.5/38.9	128.9/67.4
PFA-ADP (s)	101.0/41.8	96.5/123
PFA-EPI (s)	159.0/118.3	161.5/33.3

Abbreviations: aPPT, activated partial thromboplastin time, VWF-AG, von Willebrand factor antigen, VWF-Ac, von Willebrand activity, PFA-ADP, Platelet Function Analyzer adenosine diphosphate, PFA-EPI, Platelet Function Analyzer epinephrine.

**Table 3:** Bleeding and thrombosis score of the patient collective.

	Patient Collective (*n* = 54)	
	*n*	%
Bleeding score		
Menstruation bleeding > 4 days	4	7.4
Bleeding tendency in the family	4	7.4
Regular nosebleeds	3	5.6
Gum bleeding	2	3.7
Hematomas	1	1.9
Postoperative bleeding complications	3	5.6
Coagulation score		
Thrombosis in the family	4	7.4
Thrombosis spontaneous	1	1.9
Thrombosis after bed rest	1	1.9

**Table 4:** Differences in aPTT, VWF-Ac and factor VIII between patient and control collective (corrected for age, sex and weight).

Target Value	Coefficient	Significance	95% Confidenceinterval
aPTT	1.8 s	0.028	(0.2–3.4)
VWF-Ac	−16.4%	0.042	(−32.2–0.61)
Faktor-VIII	−28.3%	0.001	(−44.6–12.0)

Abbreviations: aPPT, activated partial thromboplastin time, VWF-Ac, von Willebrand activity.

**Table 5:** Frequency distribution of the VWS criteria in the patient and control collectives.

	Patients (*n* = 54)		Control Collective (*n* = 58)	
	*n*	%	*n*	%
VWF-Ac < 50%	5	9.3	0	0
PFA-EPI > 160 s + PFA-ADP > 121 s	7	13	8	13.8
VWF-Ac < 50% + PFA-EPI > 160 s + PFA-ADP > 121 s	4	7.4	2	3.4

Abbreviations: VWF-Ac, von Willebrand activity, PFA-EPI, Platelet Function Analyzer epinephrine, PFA-ADP, Platelet Function Analyzer adenosine diphosphate.

**Table 6:** Frequency distribution of clinical bleeding and thrombosis events in the patient collective divided into patients with and without VWS.

Patients	with VWS (*n* = 16)		without VWS (*n* = 38)	
	*n*	%	*n*	%
Bleeding	6	37.5	5	13.2
Thrombosis	1	6.3	2	5.3
Bleeding + Thrombosis	1	6.3	1	2.6
Without symptoms	8	50	30	79

Abbreviation: VWS, von Willebrand syndrome.

**Table 7:** Comparison of relevant laboratory constellations, the results of the patient questionnaires and medication history.

Lab Abnormalities													Patients	Symptoms			Relevant Medications		
PFA-Et (s)		PFA-At (s)		VWF-Ac (%)		VWF-Ag (%)		Faktor VIII (%)		aPTT (s)		without Abnormalities	(n)	Bleeding	Throm-bosis	without Symptoms	Medicament	Oral Contra-ceptive	without Medications
<84	>160	<68	>121	<50	>150	<50	>160	<70	>150	<26	>36								
	X		X										2			X			X
	X		X										1			X		X	
	X		X										1	X			Diclofenac		
	X		X										1		X				X
	X		X								X		2			X		X	
	X		X	X									1			X		X	
	X		X	X							X		1	X					X
	X		X	X		X		X					1	X	X				X
	X		X	X		X							1	X			ASS		
X		X					X				X		1			X		X	
	X												9			X			X
	X												2	X					X
	X												1		X				X
	X						X		X				1			X	SSRI		
	X			X		X							1			X			X
	X			X				X					1			X			X
	X										X		2			X			X
	X				X								1			X	ASS		
			X										2			X			X
			X							X			1			X		X	
			X	X									1	X					X
		X			X						X		1			X		X	
		X					>150		X				1	X	X			X	
				X				X			X		1	X					X
									X				1			X			X
				X							X		1	X					X
							X						1			X			X
												X	3			X	Hormones		
												X	7			X			X
												X	1		X				X
												X	2	X					X
												X	1	X			Prasugrel		

Abbreviations: aPPT, activated partial thromboplastin time, VWF-AG, von Willebrand factor antigen, VWF-Ac, von Willebrand activity, PFA-At, Platelet Function Analyzer adenosine diphosphate time, PFA-Et, Platelet Function Analyzer epinephrine time.

**Table 8:** Comparison of relevant laboratory constellations and medication history.

Lab Abnormalities								Patients	Relevant Medications		
PFA-Et (s)	PFA-At (s)	VWF-Ac (%)		Faktor VIII (%)	aPTT (s)		without Abnormalities	(n)	Medicament	Oral Contraceptive	without Medications
>160	>121	<50	>150	>150	<26	>36					
X								2	ASS		
X								1	Hormones		
X								7			X
X				X				2	ASS		
X				X				1			X
X						X		1	Hormones		
X			X					1			X
X			X	X				1	ASS		
X			X	X				1	Diclofenac		
X			X	X				1	SSRI		
X			X	X				1			X
				X				1		X	
				X				4			X
			X	X				1	Ibuprofen		
			X	X				1		X	
			X	X				2			X
			X	X	X			1			X
				X		X		1	Ibuprofen		
	X				X			1			X
	X					X		1	Ibuprofen		
	X			X				1			X
X	X							1	ASS		
X	X							3			X
X	X				X			1	ASS		
X	X					X		2			X
X	X		X	X				1	ASS		
X	X	X						1			X
X	X	X				X		1	Ibuprofen		
							X	2	Hormones		
							X	13			X

Abbreviations: aPPT, activated partial thromboplastin time, VWF-Ac, von Willebrand activity, PFA-At, Platelet Function Analyzer adenosine diphosphate time, PFA-Et, Platelet Function Analyzer epinephrine time.

**Table 9:** Frequency of use of drugs in the patient and control populations that are potential triggers for VWS, prolonged bleeding time, or thrombosis.

	Patient Collective (*n* = 54)		Control Collective (*n* = 58)	
	*n*	%	*n*	%
Drugs as possible triggers for				
Von Willebrand syndrome (VWS)	0	0	0	0
Prolonged bleeding time	5	9.3	14	25.9
Thrombosis	10	18.5	6	10.3

## Data Availability

The datasets analyzed during the current study are available from the Department of Nuclear Medicine in Mainz/Germany upon reasonable request.
